# Effect of Grain Orientation on Microstructure and Mechanical Properties of FeCoCrNi High-Entropy Alloy Produced via Laser Melting Deposition

**DOI:** 10.3390/ma16175963

**Published:** 2023-08-31

**Authors:** Fuyu Ge, Shuai Liu, Xin Zhang, Mengdie Shan, Cheng Peng, Fanghui Jia, Jian Han, Yangchuan Cai

**Affiliations:** 1 School of Materials Science and Engineering, Tianjin University of Technology, Tianjin 300384, China; 2 School of Automobile Engineering, Changshu Institute of Technology, Changshu 215500, China; 3 School of Mechanical, Materials, Mechatronic and Biomedical Engineering, University of Wollongong, Wollongong, NSW 2522, Australia

**Keywords:** high-entropy alloy, laser melting deposition, scanning strategy, mechanical property, improvement mechanism

## Abstract

The long, straight grain boundary of the high-entropy alloy (HEA) produced via laser melting deposition (LMD) is prone to cracking due to unidirectional scanning (single wall). To enhance the competitive growth of columnar grains and improve the overall performance of the alloy, a vertical cross scanning method was employed to fabricate FeCoCrNi HEA (bulk). The influence of grain orientation on the microstructure and mechanical properties of FeCoCrNi-LMD was systematically investigated. Microhardness tests and tensile tests were conducted to assess the mechanical property differences between the single-wall and bulk samples. This study shows that using a single scanning strategy results in monolayer wall grains sized at 129.40 μm, with a max texture strength of 21.29. Employing orthogonal scanning yields 61.15 μm block-like grains with a max texture strength of 11.12. Dislocation densities are 1.084 × 10^12^ m^−2^ and 1.156 × 10^12^ m^−2^, with average Schmid factors of 0.471 and 0.416. In comparison to the FeCoCrNi-LMD single wall, the bulk material produced through cross-layer orthogonal scanning exhibited reduced residual stress, weakened anisotropy, and improved mechanical properties. These findings are expected to enhance the potential applications of FeCoCrNi-LMD in various industries.

## 1. Introduction

As a novel alloy, high-entropy alloys (HEAs) have garnered significant attention due to their exceptional combination of high strength [1], ductility [1], corrosion resistance [2,3], wear resistance [4], and favorable high-temperature properties [5,6]. Due to the high-entropy effect of HEAs, these alloys have the capability to form a single-phase structure, including body-centered cubic (BCC), face-centered cubic (FCC), hexagonal close-packed (HCP), or a combination of these phases, during the solidification process. This is in contrast to the formation of complex intermetallic compounds commonly observed in traditional alloys [7,8,9]. FeCoCrNi HEA is a widely studied solid solution alloy with a face-centered cubic (FCC) crystal structure. In comparison to HEAs that exhibit a body-centered cubic (BCC) phase or a combination of BCC and FCC phases, FeCoCrNi HEA, with its single FCC phase, demonstrates good ductility. However, its strength is relatively low compared to those HEAs with a BCC or mixed-phase structure.

The traditional method for producing HEAs is vacuum arc melting, which is advantageous in terms of minimal air pollution and the ability to produce large-size metals. However, it is challenging to fabricate HEA materials with complex shapes using this method. The emergence of additive manufacturing technology, as highlighted by Lin et al. [10], addresses this limitation. Additive manufacturing, such as laser melt deposition (LMD), enables the production of HEAs with intricate geometries. Furthermore, HEAs produced by vacuum arc melting often exhibit defects such as component segregation, cold cracking, and shrinkage cavities, which significantly impact the properties of HEAs. However, research conducted by Lin et al. [10] demonstrated that the yield strength of FeCoCrNi HEA produced by LMD increased by approximately 460 MPa compared to castings. Additionally, Xiang et al. [11] found that FeCoCrNiMn HEA produced by LMD exhibited superior tensile properties compared to castings of the same alloy. In comparison to traditional manufacturing techniques, LMD offers several advantages. It enables the rapid and direct production of HEAs with complex geometric shapes [12]. The high-temperature gradient and rapid cooling rate associated with LMD technology facilitate the formation of fine microstructures and the development of HEAs with excellent overall properties [13,14]. As a result, many researchers have turned to LMD technology for the production of HEAs with enhanced mechanical properties.

In recent years, there has been a growing interest among researchers to improve the properties and microstructure of HEAs through modifications to the manufacturing processes [15,16,17]. In previous studies [18], we observed that the microstructure of the FeCoCrNi-LMD single wall consisted of coarse columnar grains. However, these coarse columnar grain boundaries were prone to cracking during the tensile process due to dislocation accumulation and stress concentration, making them weak areas in FeCoCrNi-LMD. This limitation hinders the widespread application of FeCoCrNi-LMD. Interestingly, conventional process parameters such as scanning speed, laser power, and powder feeding speed have minimal influence on texture and grain size [16,19,20,21,22,23]. Although post-processing techniques can help reduce or eliminate residual stress in parts, eliminating the residual deformation caused by plastic strain and residual stress is challenging, making it difficult to ensure the geometric accuracy of the produced parts. To address these challenges, the scanning strategy employed during the additive manufacturing process can serve as a powerful tool for controlling grain orientation and microstructure [24]. A study by Lu et al. [25] investigated 11 different scanning strategies and discovered that using an orthogonal transformation scanning method between layers can effectively reduce residual stress. Amirjan, Zhao, and Wan’s work highlighted the efficacy of orthogonal scanning in enhancing conventional alloy properties [26,27,28]. Yet, the impact of this strategy on the LMD performance of the foundational FeCoCrNi high-entropy alloy remains unexplored. This study focuses on reducing residual stresses and modifying grain growth by employing interlayer orthogonal scanning to fabricate FeCoCrNi high-entropy alloy.

The objective of this paper is to investigate a production process that can alter the grain growth orientation and analyze its impact on the mechanical properties of FeCoCrNi-LMD. Therefore, for the purpose of this study, the bulk material prepared using the same scanning method between layers was not selected for comparison. The microstructure of the bulk material obtained using the scanning method with the same direction between layers is highly similar to that of the single wall. Thus, the main focus of this research is on comparing the single wall and the bulk material prepared using the orthogonal variable direction scanning method between layers. To analyze the microstructure, super depth of field microscopy and electron backscatter diffraction (EBSD) techniques were employed. Microhardness testing and tensile tests were conducted to evaluate the mechanical properties of the materials produced using different processes. Additionally, in order to assess the material’s performance more comprehensively, a rolling process was utilized to induce continuous plastic deformation at room temperature, enabling a more direct observation of the material’s strain capability. The findings from these studies are anticipated to contribute to the advancement of FeCoCrNi-LMD’s industrial applications.

## 2. Experimental Procedures

### 2.1. Materials

**Figure 1:**
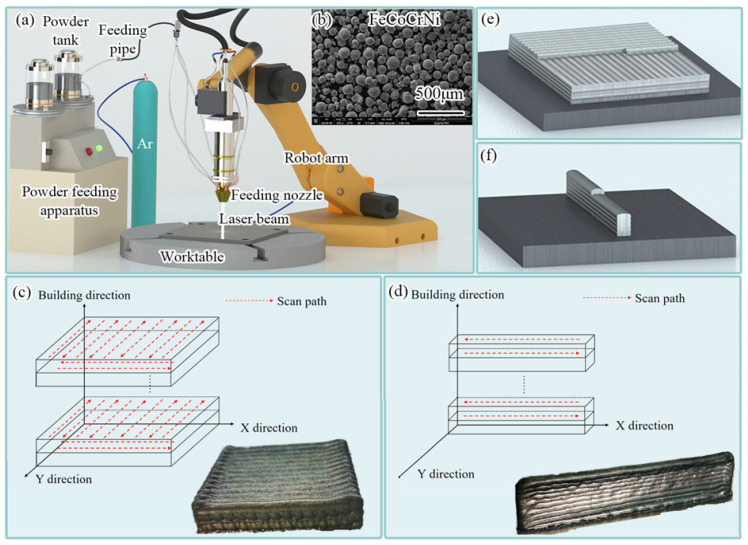
(**a**) Schematic diagram of the LMD system; (**b**) micromorphology of FeCoCrNi HEA powder; scanning mode (**c**) and product model (**e**) of bulk; scanning mode (**d**) and product model (**f**) of single wall.

The FeCoCrNi HEA was synthesized through the utilization of an LMD system (HANSGS-RJ0016-F3K, Han’s Laser Technology Industry Group Co., Ltd., Shenzhen, China), as visually represented in Figure 1a. The selection of LMD processing parameters was drawn from our comprehensive parameters database [21]: a powder feeding speed of 2.5 rpm, a laser power of 1300 W, a laser travel speed of 6 mm s^−1^, and 3 mm laser beam spot diameter. The protective gas used in the production process was Ar. Throughout the production process, the shielding gas flow rate used was kept at 5 NL min^−1^. A commercially available pre-blended powder composed of FeCoCrNi high-entropy alloy (HEA) was utilized for the manufacturing of laser metal deposition (LMD) in this study. The powder has a near-spherical morphology and a particle size in the range of approximately 45 to 150 μm, as shown in Figure 1b. The chemical composition of the HEA power is listed in Table 1. During the laser metal deposition (LMD) procedure, a foundational platform of 304 stainless steel substrate, with dimensions of 200 × 120 × 10 mm^3^ (Length × Width × Thickness), was utilized. Before the deposition process commenced, meticulous polishing and thorough cleansing were carried out on one surface of the 304 stainless steel substrate. The detailed elemental composition of the 304 stainless steel can be found in Table 2. Figure 1c–f show the scanning path and entity diagram of the bulk and single wall.

### 2.2. Microstructure Characterization and Mechanical Property

Metallographic specimens were extracted from both the singular wall and bulk regions of the manufactured FeCoCrNi-LMD material. The FeCoCrNi-LMD samples underwent grinding and standard metallographic polishing procedures. Subsequently, the polished samples were subjected to analysis utilizing the Smart Lab (Singapore) 9 KW model of X-ray diffractometer (XRD). The scan span ranged from 20° to 100° (2θ) at a scanning rate of 3° per minute. Following this, the polished samples were immersed in aqua regia (consisting of nitric acid and hydrochloric acid in a 1:3 ratio) for a duration of approximately 15 s. The DSX-HRUF ultra-depth field microscope was employed to observe the macroscopic structure of the cross-sectional morphology.

Using the TESCAN MAIA3 apparatus (Shanghai, China) equipped with Channel 5 software, an electron backscatter diffraction (EBSD) analysis was executed. The EBSD specimens were meticulously chosen with step sizes of 4 μm and 6 μm, all maintained at room temperature. To alleviate potential strain arising from sample preparation, ion etching was applied, operating at a 6.5 V voltage for a duration of 30 min. Leveraging the FEI TECNAI F30 transmission (Hillsboro, OR, USA) electron microscope (TEM), intricate electron images at high magnification were captured, along with obtaining selected area diffraction patterns.

**Figure 2:**
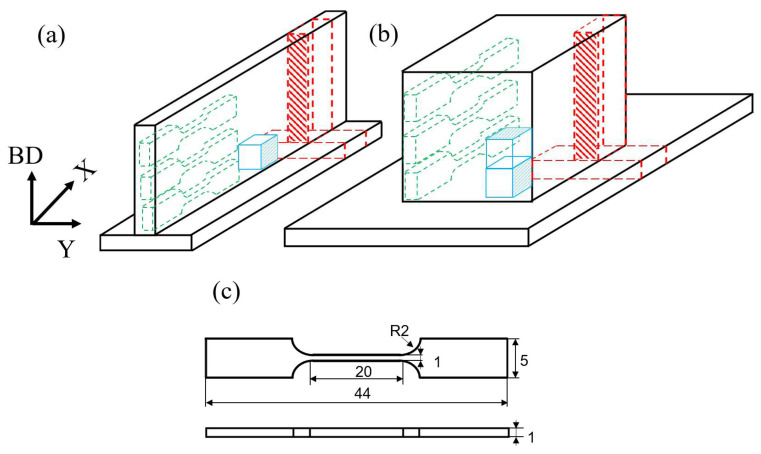
Schematic diagram of sample location and size: (**a**) sampling location of the single wall; (**b**) sampling location of the bulk; (**c**) tensile sample (in mm).

The microhardness of the HEA sample was assessed using the HMV-2T microhardness tester. Microhardness tests were conducted at intervals of 1 mm. To apply tension to the material at a rate of 1 mm per minute, a WDW-300C universal testing machine (Shandong, China) was employed. The fracture of post-test tensile samples was observed using the Quanta FEG 250 scanning electron microscope (SEM)(FEI, Columbia, MD, USA). The sampling positions are depicted in Figure 2a,b, with the shaded portion indicating the selected testing plane. The dimensions of the tensile specimen are illustrated in Figure 2c.

**Figure 3:**
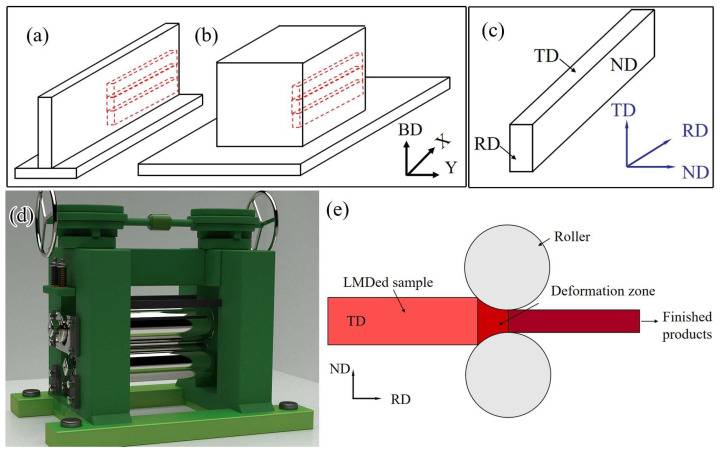
Sampling location for rolled specimens of (**a**) single wall and (**b**) bulk; (**c**) schematic diagram of rolling direction; (**d**) schematic diagram of cold rolling mill; (**e**) schematic diagram of rolling process.

In this study, a rolling process was employed to induce continuous plastic deformation in the samples. Prior to rolling, the samples underwent a cleaning process using an ultrasonic cleaner. The rolling was performed using a Φ300 × 350 two-roll cold rolling mill. The dimensions of the samples were 50 × 5 × 2 mm^3^ (Length × Width × Thickness). The rolling process involved incrementally applying rolling deformation at 0%, 25%, and 50% increments. The rolling speed was set at 5 m per minute. Figure 3 illustrates the schematic diagram of the two-roll cold rolling mill and the rolling process. In the diagram, BD indicates the direction of addition, RD represents the rolling direction, TD stands for the cross-section, and ND refers to the normal direction.

## 3. Results and Discussion

### 3.1. Microstructure of the FeCoCrNi HEA

**Figure 4:**
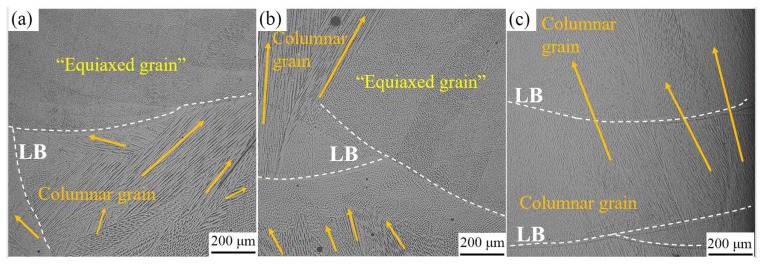
Microstructure of FeCoCrNi-LMD bulk and single wall: (**a**) X-Z plane of bulk; (**b**) X-Y plane of bulk; (**c**) X-Z plane of single wall.(The orange arrow represents the growth direction of the columnar crystal.)

In preceding studies [18], our research team uncovered a persistent columnar grain growth pattern within the single wall of FeCoCrNi-LMD, extending seamlessly across multiple layers from bottom to top. This growth led to the formation of equiaxed grains in the uppermost layer. Figure 4 visually presents the microstructure of both the FeCoCrNi-LMD bulk and the single wall. Of significance, the columnar grains in the single wall demonstrate continuous expansion across the layer boundary (LB). Unlike the single wall, the epitaxial growth of columnar grains in the bulk was not prominent, and ‘equiaxed grains’ can also be seen in the cross-section morphology. The XY plane of the bulk also has obvious columnar grains and ‘equiaxed grains’, which indicates that these grains may be located in the growth direction close to the normal direction of the cross-section, thus showing the growth cross-section of the grains themselves. Whether it was equiaxed grain or grain growth cross-section observed in bulk metallography, it showed that the grain growth process in bulk was quite different from that in the single wall. By comparison, we can find that the grain number in the bulk was much more than that in the single wall, and the grain morphology was more different than that in the single wall. When the layers were the same, the heat accumulation of the bulk was significantly higher than that of the single wall. Moreover, due to the change in the scanning path, the direction of heat flow also changed. Shifting the heat flux direction and adjusting heat accumulation cause shifts in the temperature gradient (G), cooling rate (ε), and growth rate (V). An elevated temperature gradient (G), cooling rate (ε), and growth rate (V) have the capacity to alter the solidification behavior of metallic materials [29].

**Figure 5:**
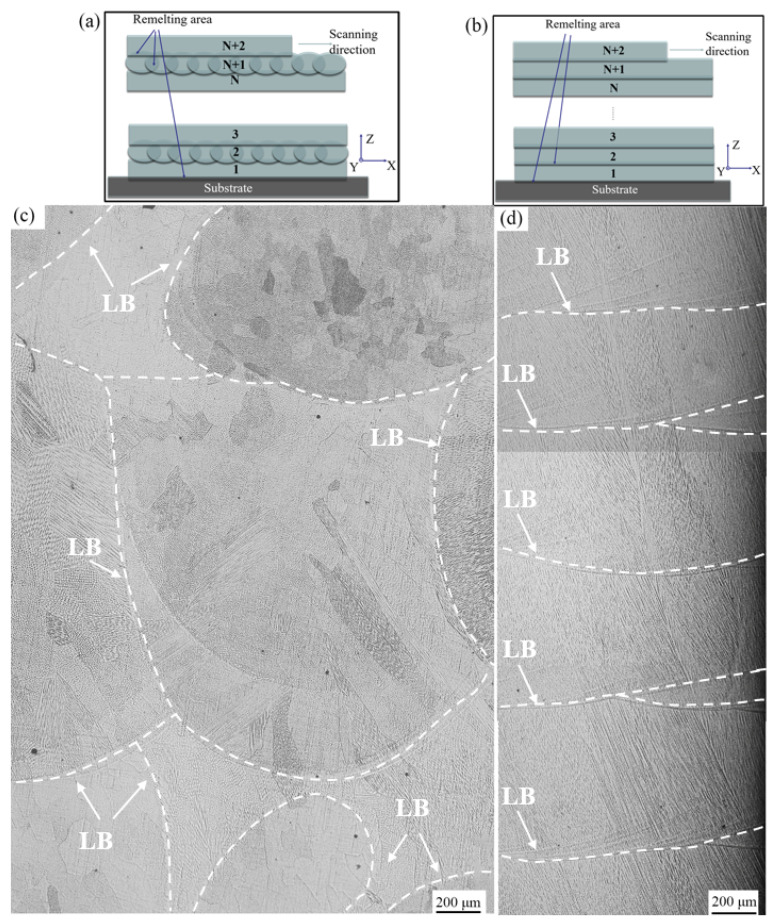
The scanning path diagram and multilayer microstructure of FeCoCrNi-LMD bulk and single wall: (**a**) scanning path diagram and (**c**) multilayer microstructure of bulk; (**b**) scanning path diagram and (**d**) multilayer microstructure of single wall.

Figure 5 illustrates the scanning path diagram and multilayer microstructure of the FeCoCrNi-LMD bulk material and single wall. As shown in Figure 5a,b, during laser scanning in the N layer (N ≥ 2), there are differences in the smoothness between the bulk material and the single wall. This leads to variations in the remelted regions and liquid flow directions in each layer. In the bulk material, there is overlap between adjacent layers on the X-Y plane, which introduces changes in the temperature field within the molten pool. Figure 5 illustrates the scanning path diagram and multilayer microstructure of the FeCoCrNi-LMD bulk material and single wall. As shown in Figure 5a,b, during laser scanning in the N layer (N ≥ 2), there are differences in the smoothness between the bulk material and the single wall. This leads to variations in the remelted regions and liquid flow directions in each layer. In the bulk material, there is overlap between adjacent layers on the X-Y plane, which introduces changes in the temperature field within the molten pool.

**Figure 6:**
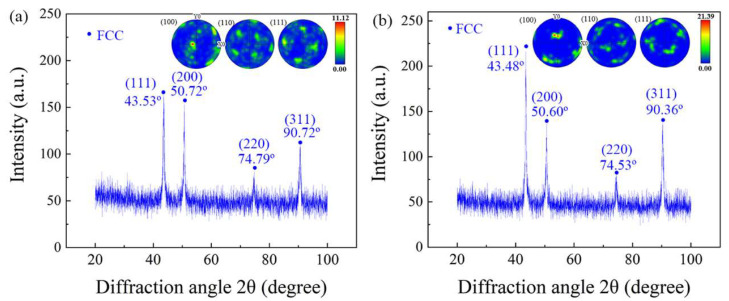
EBSD-pole figure and XRD of FeCoCrNi-LMD bulk and single wall: (**a**) bulk; (**b**) single wall.

The EBSD-pole figure and XRD analysis of both the FeCoCrNi-LMD bulk material and single wall are illustrated in Figure 6. The EBSD-pole figure unveils a cubic texture marked by a <001>//BD orientation. The [001] direction aligns with the deposition direction. In the process of laser deposition, the laser beam strikes the molten pool perpendicularly, concentrating energy at its upper section. As a result, a significant vertical temperature gradient forms within the molten pool. Heat disperses predominantly in the vertical direction, exerting influence on the growth of columnar grains along the path of the highest temperature gradient. Consequently, the initial texture of both the FeCoCrNi-LMD bulk material and single wall showcases a <001>//BD columnar grain arrangement [30].

The single wall exhibits a strong texture, with a maximum pole density of 20.33. In contrast, the bulk material has a texture density of 11.12, which is 45.30% lower than that of the single wall. This suggests that the interlayer orthogonal scanning method employed during the production of FeCoCrNi-LMD promotes the formation of specific or random orientations, resulting in a weakened maximum texture density.

According to the XRD patterns of FeCoCrNi-LMD bulk and single wall, both of them are FCC, which is attributed to the high-entropy effect of HEA. In the crystal material, when the crystal structure of the tested sample presents different angles with the incident X-ray and conforms to the Bragg lattice equation (2dsin⁡θ=nλ), the X-ray on the crystal plane with spacing *d* is incident at a certain incident angle *θ*, and the superposition diffraction peak results will appear in the diffraction pattern. For the XRD results in this experiment, both are FCC phase and belong to the cubic crystal system. Therefore, the calculation method of crystal plane spacing *d* is as follows:

(1) $$d=a/{\left(h^{2}+k^{2}+l^{2}\right)}^{1/2}$$

The calculation method of lattice constant a is as follows:

(2) $$a=\lambda {\left(h^{2}+k^{2}+l^{2}\right)}^{\frac{1}{2}}/(2\sin\theta )$$

where *h*, *k*, and *l* are the crystal plane indices corresponding to the diffraction peak, *θ* is the diffraction angle, and *λ* is the wavelength of X-ray (0.154056 nm), respectively. When Equation (2) was used to calculate the lattice constant of the crystal, the selection of high-angle diffraction peaks will increase the accuracy of the results. Therefore, the diffraction angle corresponding to (311) was selected for calculation in this experiment. The lattice constant of the bulk was 0.3602 nm, and that of the single wall was 0.3590 nm.

**Figure 7:**
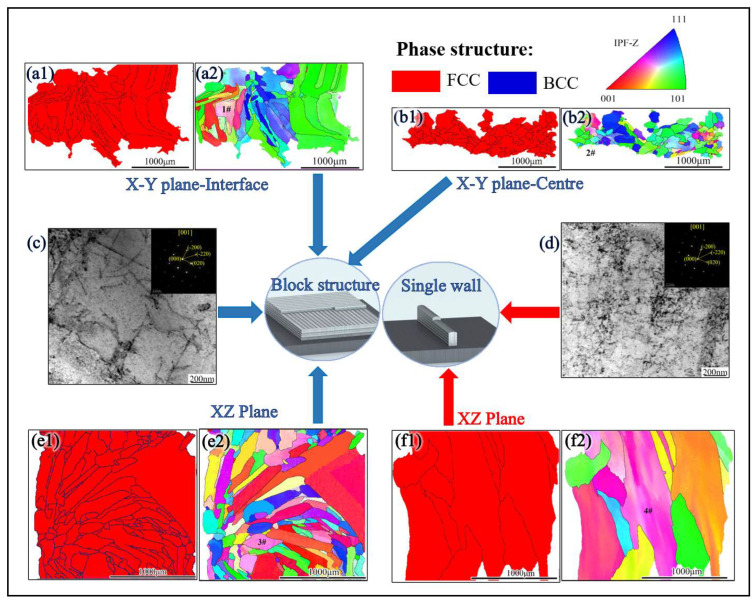
EBSD-phase structure, EBSD-IPF, and TEM analysis of FeCoCrNi-LMD: EBSD-phase structure (**a1**) and EBSD-IPF (**a2**) X-Y plane-interface of bulk; EBSD-phase structure (**b1**) and EBSD-IPF (**b2**) X-Y plane-center of bulk; (**c**) the bright-field image and electron diffraction spots of the bulk; (**d**) the bright-field image and electron diffraction spots of the single wall; EBSD-phase structure (**e1**) and EBSD-IPF (**e2**) X-Z plane of bulk, (**d**) X-Z plane of single wall, EBSD-phase structure, (**f1**) and EBSD-IPF (**f2**) X-Z plane of single wall.

Figure 7 illustrates the EBSD-phase structure, EBSD-inverse pole figure (IPF), and TEM analysis of FeCoCrNi-LMD bulk and single walls. To analyze the difference between the X-Y lap joint and the non-lap joint in the bulk material, the grains were divided into two categories based on their position using the post-processing function of EBSD. One category consists of grains located at the overlap of the bulk X-Y plane, as shown in Figure 7(a1,b1). The second category encompasses grains positioned outside the bulk X-Y plane, as illustrated in Figure 7(a2,b2). In Figure 7(a1,b1,e1,f1), the phase structure is presented across various planes: the X-Y plane at the bulk material interface, the X-Y plane at the bulk material center, the X-Z plane of the bulk material, and the X-Z plane of the single wall. In the EBSD-phase structure, the FCC phase is highlighted in red, while the BCC phase is depicted in blue. Notably, both the bulk material and the single wall exhibit an FCC phase. Turning to Figure 7c,d, these images display the bright-field image and electron diffraction spots of the FeCoCrNi-LMD material. The presence of FCC phase is indicated by the electron diffraction spots in both images. This confirms that alterations in the scanning strategy do not impact the phase structure of FeCoCrNi-LMD.

The EBSD-inverse pole figure (IPF) maps of the X-Y plane at the interface of the bulk material, the X-Y plane at the center of the bulk material, the X-Z plane of the bulk material, and the X-Z plane of the single wall are presented in Figure 7(a2,b2,e2,f2). The internal color of the grains at positions 1#, 2#, and 3# has changed, primarily due to the ability of EBSD to measure the difference in grain orientation. The grain boundaries with an orientation difference of 15° were used as a criterion for grain division. As a result, most of the larger grains exhibit the same orientation.

**Figure 8:**
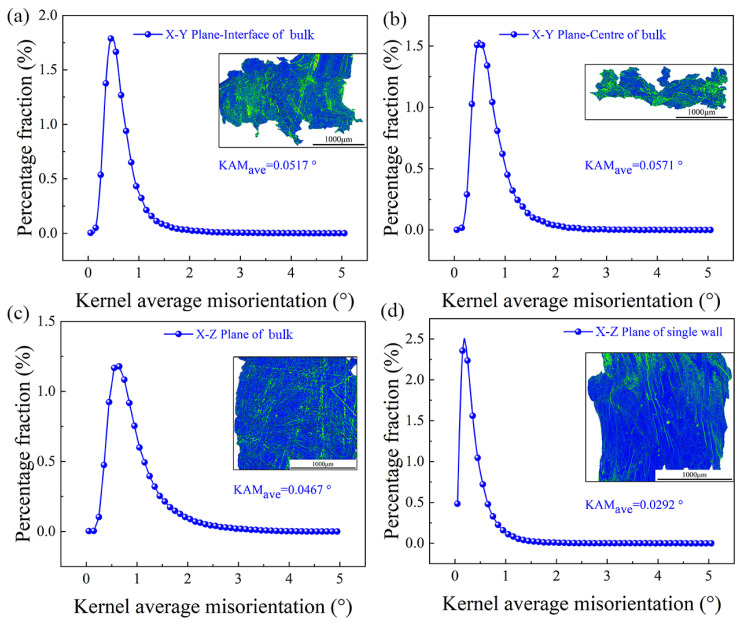
EBSD-KAM distribution curve and EBSD-KAM of the FeCoCrNi-LMD: (**a**) X-Y plane-interface of bulk; (**b**) X-Y plane-center of bulk, (**c**) X-Z plane of bulk, (**d**) X-Z plane of single wall.

Figure 8 depicts the EBSD-kernel average misorientation (KAM) and EBSD-KAM distribution curve for both the FeCoCrNi-LMD bulk and single wall. The graph’s green lines indicate geometrically necessary dislocations, essentially representing dislocations. Notably, both the bulk and single wall exhibit a pronounced dislocation density due to the rapid solidification rates inherent in LMD [11]. During the additive manufacturing process, as the metal quickly solidified and cooled from high temperatures, a multitude of supersaturated vacancies formed within the grains, coalescing to form dislocations [29]. Especially noteworthy, the number of dislocations observed on the X-Y and X-Z planes of the bulk surpasses those on the X-Z plane of the single wall. The EBSD analysis suggests that the bulk possesses smaller grain sizes compared to the single wall, consequently leading to an increased count of grain boundaries within the bulk. Near these boundaries, the interplay of thermal and organizational stresses often triggers stress concentration. Whenever the stress reaches a critical point, localized slipping occurs, giving rise to the generation of dislocations within that region [29]. Therefore, the smaller the grain size of HEA and the more grain boundaries there are, the greater the dislocation density.

Within this research, the investigation into the progression and concentration of geometrically necessary dislocations (GNDs) was conducted through the utilization of the local orientation difference method. Here, KAM_ave_ symbolizes the weighted mean of the KAM values. The determination of dislocation density was performed using EBSD data, employing the equation denoted as (3), the average value of KAM can be used to quantitatively calculate the GND density ($\rho^{GND}$) [31,32]:

(3) $$\rho^{GND}=\frac{2{KAM}_{ave}}{\mu b}$$

where μ is the step size (4 μm and 6 μm) and b is the Burgers vector (2.35 × 10^−10^). According to the KAM_ave_ value of bulk and single wall, the dislocation density of the X-Y plane-interface (μ = 6 μm) of bulk was 1.280 × 10^12^ m^−2^, the dislocation density of X-Y plane-center (μ = 6 μm) of bulk was 1.414 × 10^12^ m^−2^, and the dislocation density of X-Z plane (μ = 6 μm) of bulk was 1.156 × 10^12^ m^−2^. The dislocation density of the single-wall X-Z plane (μ = 4 μm) was 1.084 × 10^12^ m^−2^. The dislocation density of bulk X-Z plane was slightly higher than that of single-wall X-Z plane, but the difference was not large, which can be seen from EBSD-KAM.

**Figure 9:**
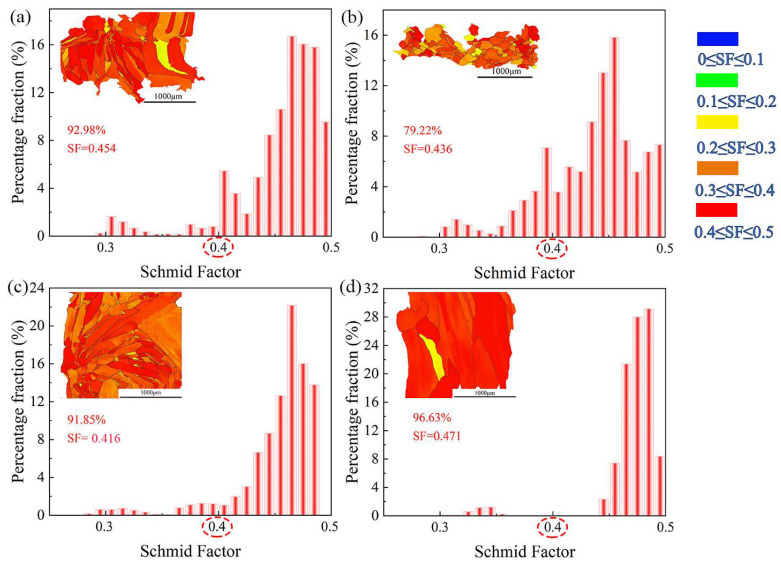
EBSD-SF distribution diagram and EBSD-SF of the FeCoCrNi-LMD: (**a**) X-Y plane-interface of bulk; (**b**) X-Y plane-center of bulk, (**c**) X-Z plane of bulk, (**d**) X-Z plane of single wall.

Figure 9 shows the EBSD-SF distribution diagram and EBSD-SF of FeCoCrNi-LMD bulk and single wall. The shear stress *τ* resulting from the external force *F* applied along the slip direction within the slip plane can be expressed as [29]:

(4) $$\tau =\frac{F}{A}\cos\varphi \cos\lambda$$

where cos⁡φcos⁡λ is the orientation factor or SF. The investigation carried out by Gussevet et al. [33] revealed that the Schmid factor (SF) exceeds 0.4 for soft orientations, while it drops below 0.35 for hard orientations. A higher orientation factor indicates an increased likelihood of grain deformation, facilitating its occurrence. Conversely, a lower orientation factor signifies heightened resistance to grain deformation, making the process more intricate to initiate. In Figure 9d, the grain structure was mostly covered by red, indicating that the SF of the single wall was larger than bulk, and the probability of slip was also larger than bulk. The grain structure in Figure 9a–c was covered in orange, which indicates that the SF of the X-Y lap joint was smaller than that of the non-lap joint grain and the probability of slip was lower than that of the non-lap joint grain.

Based on the EBSD-SF distribution diagram, the average stacking fault (SF) value in the single wall along the X-Z plane was 0.471, and the proportion of soft grains was 96.63%. This indicates that the X-Z plane of the single wall is more susceptible to deformation. In the bulk material, the average SF value in the X-Y plane at the interface was 0.454, and the proportion of soft grains was 92.98%, which is similar to the single-wall X-Z plane. However, in the X-Y plane at the center of the bulk material, the average SF value was 0.436, and the proportion of soft grains was 79.22%. The grain plasticity in the X-Y plane lap joint was higher than that in the non-lap joint. The overlapping of bulk material in the X-Y plane improved the plasticity of the material. Overall, these observations indicate that the X-Y plane of the bulk material, particularly at the center, exhibits higher grain plasticity compared to the single wall. The lap joint configuration in the X-Y plane enhances the material’s plasticity.

### 3.2. Mechanical Properties of Bulk and Single Wall

**Figure 10:**
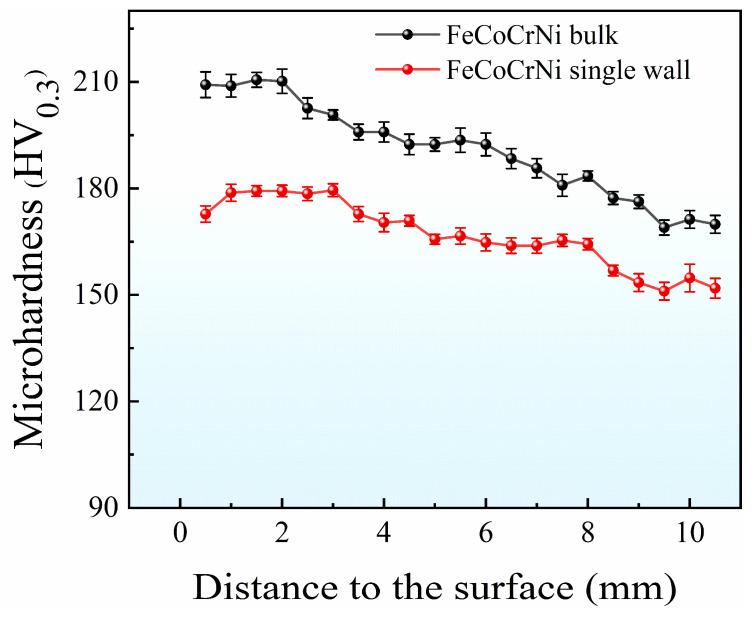
Microhardness test results of the FeCoCrNi-LMD.

Figure 10 illustrates the results of the microhardness test conducted on FeCoCrNi-LMD samples. The highest microhardness value observed in the bulk material was 210.6 HV_0.3_, while the highest microhardness value in the single wall was 180.5 HV_0.3_. The general trend depicted in the results indicates that the microhardness value decreases with an increase in the number of layers. When comparing materials produced under the same process parameters, the bulk material exhibited higher hardness than the single wall. The difference in microhardness can be primarily attributed to the average grain size and the original dislocation density of the material. According to the Hall–Petch formula, smaller grain sizes correspond to higher hardness values. Moreover, the dislocation density also influences the material’s microhardness. Although the EBSD results indicate some variation in dislocation density, the difference was relatively small, resulting in a relatively weak impact on dislocation strengthening. The significant increase in hardness observed is primarily attributed to the process of grain refinement [34].

**Figure 11:**
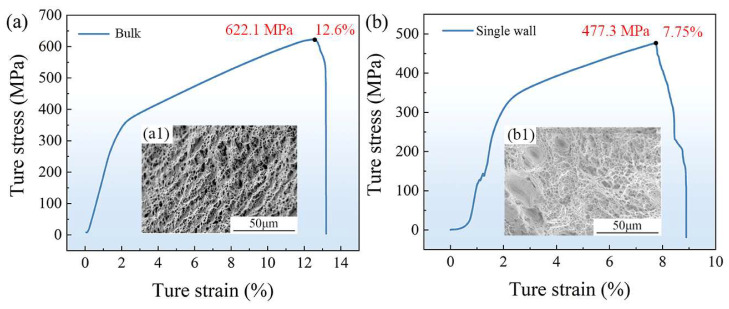
Tensile test results and fracture micro morphology of FeCoCrNi-LMD: (**a**) stress–strain curve and (**a1**) microstructure of fracture surface of the bulk; (**b**) stress–strain curve and (**b1**) microstructure of fracture surface of the bulk.

Figure 11 depicts the results of the tensile test and the fracture micromorphology of FeCoCrNi-LMD. The bulk material exhibited higher yield strength, tensile strength, and ductility compared to the single wall. The fracture surface of the single-wall specimen displayed large and deep equiaxed dimples. In contrast, the fracture surface of the bulk material exhibited micro-holes and small dimples, indicative of typical microporous ductile fracture characteristics. The fractures observed in the tensile process were primarily attributed to the presence of internal dislocations. Dislocations accumulated at grain boundaries and defects during the tensile process, leading to the initiation and growth of micro-voids at stress concentrations. This process ultimately resulted in the necking and fracture of the material.

**Figure 12:**
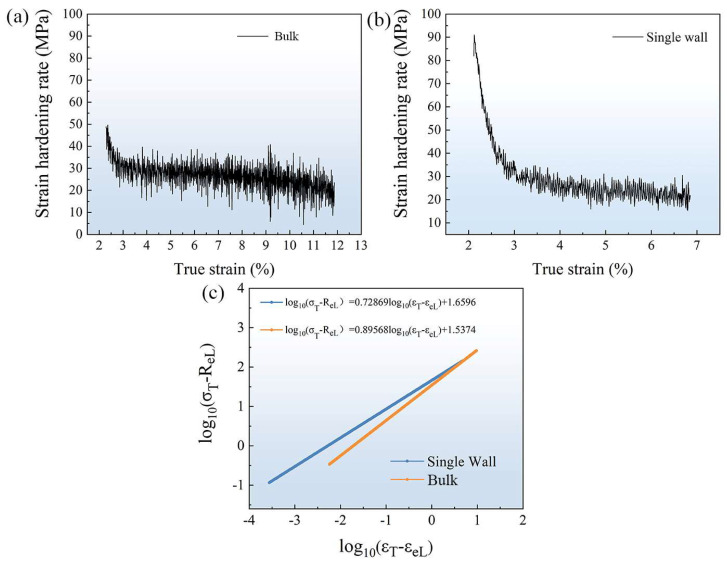
Strain hardening rate and strain hardening exponent curves of the FeCoCrNi-LMD: (**a**) strain hardening rate of bulk; (**b**) strain hardening rate of single wall; (**c**) strain hardening exponent curve of FeCoCrNi-LMD.

The rise in dislocation density, coupled with its role in impeding plastic deformation, led to the phenomenon known as work hardening [29]. Within the context of this study, the segment of the true stress–strain curve associated with work hardening underwent fitting analysis to determine the strain hardening exponent (*n*) for the reactive metal material subjected to uniform plastic deformation. The strain hardening rate profiles for FeCoCrNi-LMD are illustrated in Figure 12a,b. Figure 12c presents the strain hardening exponent curves corresponding to the work hardening segments displayed in Figure 12a,b. During the phase of uniform plastic deformation, the flow stress conforms to the Hollomon relation [35]:

(5) $$\sigma_{T}=K\varepsilon_{T}^{n}$$

where *n* is the strain (or work) hardening exponent, *K* is the strength coefficient, $\sigma_{T}$ is the true stress, and $\varepsilon_{T}^{n}$ is the true strain. The arrangement can be obtained by Equation (6):

(6) $$\log_{10}\left(\sigma_{T}-R_{eL}\right)=\log_{10}K+n\log_{10}\left(\varepsilon_{T}-\varepsilon_{eL}\right)$$

where $R_{eL}$ and $\varepsilon_{eL}$ denote d yield stress and yield strain, respectively. According to the fitting results, the strain hardening index *n* of the bulk was higher than that of the single wall. The findings demonstrated that in comparison to the single wall, the bulk material displayed a prominent strain hardening effect. This heightened strain hardening capacity of the processed components enhances their ability to withstand accidental overloads during service. This attribute plays a crucial role in preventing persistent plastic deformation in weaker areas, ensuring the material’s safe and reliable performance over its operational lifespan.

**Figure 13:**
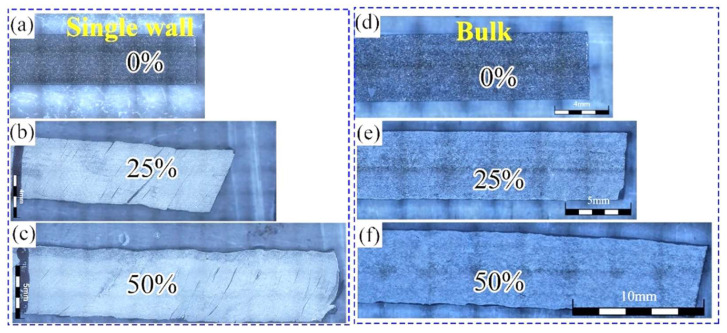
Morphology of FeCoCrNi-LMD single wall (**a**–**c**) and bulk (**d**–**f**).

Figure 13 displays the macroscopic morphologies of FeCoCrNi-LMD single walls and bulk materials with varying rolling deformations. Even at low levels of rolling deformation, noticeable cracks are observed in the single walls, and these cracks exhibit similar angles. However, the bulk materials prepared using the orthogonal bidirectional scanning strategy do not exhibit any cracks throughout the rolling process. This observation suggests that modifying the scanning process can effectively prevent crack formation in FeCoCrNi-LMD during its use.

**Figure 14:**
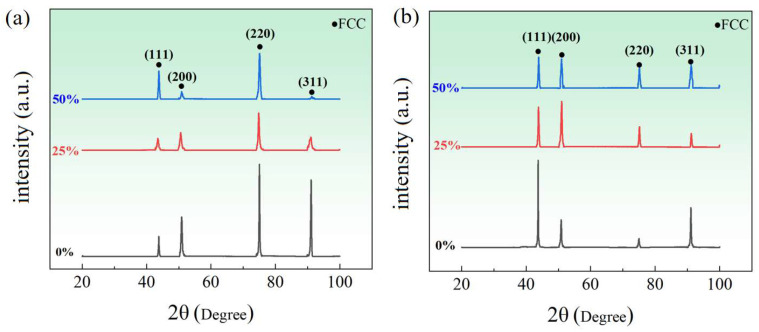
XRD patterns of FeCoCrNi-LMD bulk and single wall with different rolling deformation: (**a**) bulk; (**b**) single wall.

Figure 14 illustrates the XRD spectra of FeCoCrNi-LMD bulk materials and single walls. No structural changes were observed in the phase structure after the rolling process, indicating the stability of the FCC phase structure. This stability can be attributed to the unique sluggish diffusion effect present in high-entropy alloys. In high-entropy alloys, the inclusion of multiple elements with substantial differences in atomic radii leads to pronounced lattice distortions within the microstructure. The complex internal structure of high-entropy alloy systems makes atomic diffusion more difficult, ultimately contributing to the stability of the phase structure.

## 4. Discussion

Using Equation (2), the lattice constant of the block material is calculated to be slightly higher than that of the single wall, indicating that the lattice distortion in the single wall is greater than in the block. This suggests that the residual stress in the single wall is higher compared to the block material. This result can be attributed to the higher number of annealing cycles during the preparation process of the block material compared to the single wall. Consequently, the block material has lower residual stress than the single wall, leading to a slightly higher lattice constant in the block. Furthermore, both the single wall and the block material exhibit numerous (001) and (101) oriented grains on the X-Z plane. This alignment is due to the grain growth orientation being consistent with the direction of the maximum temperature gradient within the melt pool. However, during the production of the block material, disturbances occur on both sides of the dendrite due to variations in local heat flow. These disturbances promote dendritic growth along orthogonal directions, altering the growth mode and causing deviations from the preferred orientation [36]. Consequently, grains oriented in the (111) direction are present on the X-Z surface of the block material. The presence of multiple grain orientations in the block material indirectly indicates a reduction in anisotropy compared to the single wall. The orientation of the grains directly influences the stress state under external forces.

When external forces are applied to polycrystalline materials, the stress experienced by individual grains of varying orientations is non-uniform due to the anisotropy of the crystals. Significant disparities in shear stresses arise along different slip systems based on the grain orientations. As a result, not all grains deform simultaneously, and those with favorable slip orientations undergo deformation first. Moreover, grains with different orientations possess distinct slip directions, preventing direct propagation of slip from one grain to another. Nevertheless, in a polycrystalline material, each individual grain is surrounded by neighboring grains, and its deformation must be coordinated with the deformation of adjacent grains to maintain grain continuity. Failure to achieve such coordination can make deformation challenging and may even result in the loss of grain continuity, leading to the formation of voids and ultimately material fracture [29]. In polycrystalline materials, achieving coordination and cooperative deformation between grains requires more than just slip along the most favorable individual slip system. Each grain must also undergo slip on multiple slip systems, including those with less favorable orientations, resulting in corresponding shape changes. When highly textured materials (such as single walls) are subjected to external loads, the consistency of crystal orientations leads to the concentration of shear stresses across grain boundaries in specific directions, which can result in fracture. In contrast, materials with a weak texture (such as block materials) exhibit a random distribution of crystal orientations, leading to a more uniform stress distribution across grain boundaries. This makes them less prone to stress concentration in specific directions and gives them higher fracture toughness. Therefore, materials with a strong texture are more susceptible to fracture under external loads, whereas materials with a weak texture exhibit greater resistance and are less prone to fracture. As a result, in the rolling process, the single wall is more prone to early cracking compared to the block material. Furthermore, plastic deformation occurring during the rolling process results in the proliferation of internal dislocations within grains. The distribution of dislocations in the single wall is uneven, with localized regions exhibiting a higher dislocation density. Under stress, localized regions are susceptible to severe pile-up and the entanglement of dislocations, impeding their motion and leading to the formation of “Frank-Read dislocation sources.” This promotes the proliferation of dislocations. As a result, the dislocation density in the single wall after rolling is higher compared to that in the block material. In contrast, the distribution of dislocations in the block material is more uniform, leading to a lower dislocation density compared to the single wall. This difference in dislocation distribution and density is also a significant factor contributing to the formation of cracks [37].

**Figure 15:**
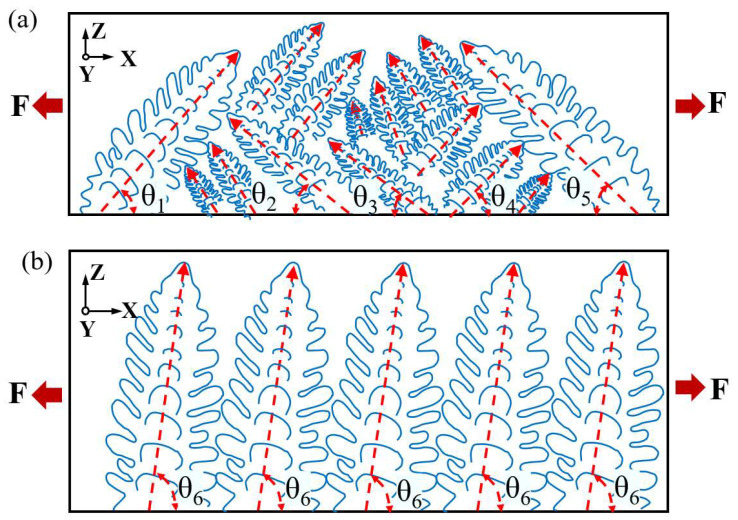
Stress diagram of grain during stretching: (**a**) bulk; (**b**) single wall.(The red arrow represents the direction of grain growth.)

To provide a more precise description of the stress state at the microstructural level for a block and a single wall, Figure 15a,b depict the stress diagrams of grains during a tensile test. The grain orientation within the bulk material appears relatively dispersed, whereas in the single wall, it appears relatively aligned. Consequently, the anisotropy of the block material is weaker compared to that of the single wall. When an external force F is applied to the material, the component force acting perpendicular to the direction of grain growth in the single wall is $F\sin\theta_{6}$, where $\theta_{6}$ is close to 90°. Therefore, the component force of the external force F acting perpendicular to the grain growth direction in the single wall is close to F. The component force acting perpendicular to the grain growth direction in the bulk material is Fsin⁡θ, but the difference in its magnitude is significant. Overall, when the same force F is applied, the load borne by the bulk material is lower than that of the single wall. Consequently, the tensile strength of the bulk material is higher compared to that of the single wall.

The block material exhibits higher microhardness, yield strength, and strain hardening compared to the single structure. This can be attributed to several strengthening mechanisms within the material’s microstructure, including solid solution strengthening, grain boundary strengthening, and dislocation strengthening. However, since both materials are FCC phases, second-phase strengthening is not a contributing factor. According to reports [38,39], solid solution strengthening is influenced by interactions between solid solutions and differences in atomic size. However, both factors are determined by the composition and content of the elements rather than the processing technique. Therefore, the effects of solid solution strengthening and second-phase strengthening can be disregarded, and the focus should primarily be on grain boundary strengthening and dislocation strengthening. Thus, the improved mechanical properties of the block material compared to the single structure can be mainly attributed to dislocation strengthening and grain boundary strengthening resulting from grain refinement. In the rolling experiment, the enhanced strain hardening capability can be attributed to the disruption of grain orientation caused by the change in scanning mode.

## 5. Conclusions

(1). The bulk structure of FeCoCrNi-LMD exhibits higher microhardness, tensile strength, ductility, and strain hardening exponent in comparison to the single-wall structure. These findings suggest that altering the scanning pattern leads to enhanced mechanical properties of FeCoCrNi-LMD.
(2). The alteration in the scanning strategy has effectively improved the weak area. The single wall, subjected to a rolling deformation of 25%, exhibited cracking, whereas the bulk material remained crack-free even at a deformation level of 50%.
(3). In comparison to the single-wall structure, the bulk structure displays a more uniform distribution of dislocations and lower dislocation density. This disparity serves as one of the primary reasons for the increased susceptibility of the single wall to cracking.
(4). In the single-wall structure, grains tend to grow along the additive direction, resulting in elevated texture strength and notable anisotropy. However, during plastic deformation, there is inadequate coordination among grains, and the continuity between them cannot be sustained. Consequently, the material is prone to the formation of voids at grain boundaries, ultimately leading to cracking.

## Figures and Tables

**Table 1:** Chemical composition of FeCoCrNi.

Element	Cr	Fe	Ni	Co
wt.%	22.60	24.05	25.93	Bal.
at%	24.52	24.30	24.93	Bal.

**Table 2:** Chemical composition of 304 stainless steel.

Element	Cr	Ni	Mn	Si	Co	N	C	Fe
wt.%	19.90	7.96	0.98	0.30	0.21	0.09	0.05	Bal.

## Data Availability

The data of this paper can be provided on reasonable request.
